# Lymphoblastoid and *Jurkat* cell lines are useful surrogate in developing a CRISPR-Cas9 method to correct leukocyte adhesion deficiency genomic defect

**DOI:** 10.3389/fbioe.2025.1548227

**Published:** 2025-03-21

**Authors:** Ahmad R. Ramadan, Noureddine Ben Khalaf, Khaled Trabelsi, Halla Bakheit, Imen Ben-Mustapha, Mohamed-Ridha Barbouche, M-Dahmani Fathallah

**Affiliations:** ^1^ Laboratory of Transmission, Control and Immunobiology of Infections (LR16IPT02), Institut Pasteur de Tunis, University of Tunis El Manar, Tunis, Tunisia; ^2^ Department of Life Sciences, King Fahd Chair of Medical Biotechnology, College of Graduate studies Arabian Gulf University, Manama, Bahrain; ^3^ Department of Molecular Medicine, College of Medicine and Health Sciences, Arabian Gulf University, Manama, Bahrain; ^4^ Biotechnology Development Group, Laboratory of Molecular Microbiology, Vaccinology and Biotechnology Development, Institut Pasteur de Tunis, University of Tunis El Manar, Tunis, Tunisia; ^5^ Faculty of Medicine, University of Tunis El Manar, Tunis, Tunisia; ^6^ Department of Microbiology, Immunology and Infectious Diseases, College of Medicine and Health Sciences, Arabian Gulf University, Manama, Bahrain

**Keywords:** leukocyte adhesion deficiency type one (LAD1), CRISPR-Cas9, ITGB2 gene, GRNA, *Jurkat* cell line, HDR

## Abstract

**Introduction:** Leukocyte adhesion deficiency type 1 (LAD1) is a severe inborn error of immunity caused by mutations in the ITGB2 gene, which encodes the beta-2 integrin subunit (CD18). These mutations lead to the absence or deficiency of CD18/CD11a, b, and c heterodimers, crucial for leukocyte adhesion and immune function. CRISPR-Cas9 Gene editing technology represents a promising approach for correcting these genomic defects restore the stable expression of CD18 and reverse the disease.

**Methods:** We developed a CRISPR-Cas9-based gene correction strategy using *Jurkat* cells and patient-derived lymphoblastoid cell lines as surrogates for hematopoietic progenitor cells. Three candidate gRNAs were first predicted in silico using CRISPOR and experimentally tested in wild-type ITGB2-expressing *Jurkat* cells to identify the gRNA with the highest genomic DNA cleavage efficiency. The most efficient gRNA was then paired with espCas9 and used alongside five homology-directed repair templates (HDRs) (single-stranded donor oligonucleotides, ssODNs) to repair ITGB2 defects in patient-derived lymphoblastoid cell lines. CD18 expression levels in edited cells were quantified via flow cytometry, and whole-genome sequencing (WGS) was conducted to assess off-target effects and insertion accuracy.

**Results:** Among the three candidate gRNAs, 2-rev gRNA exhibited the highest genomic cleavage rate in *Jurkat* cells. Using this gRNA with espCas9 and HDR-2, we achieved a 23% restoration of CD18 expression in LAD1 patient-derived cells, a level sufficient to change the disease course from severe to moderate. Whole-genome sequencing confirmed the absence of off-target mutations or undesired DNA insertions, demonstrating high specificity and precision in gene correction.

**Discussion:** This CRISPR-Cas9-based method provides a precise and effective approach for correcting ITGB2 mutations in LAD1 patients. The high-fidelity gene editing process, validated through WGS, supports its potential for future applications in CD34+ hematopoietic stem cell therapies. The approach can be further optimized for clinical translation, offering a path toward a stable and long-term cure for LAD1.

## 1 Introduction

Leukocyte adhesion deficiency (LAD1) is a rare autosomal recessive genetic disorder. The diagnosis of this inborn error of immunity is based on the clinical observations of delayed umbilical cord separation, recurrent life-threatening bacterial and fungal infections, impaired pus formation, poor wound healing, and persistent leukocytosis (Arnaout et al., 1982; Anderson and Springer, 1987; Arnaout, 1990; Wada et al., 2011). Variants of LAD1 have also been reported (Kuijpers et al., 1997; Cabanillas et al., 2016; Moutsopoulos et al., 2017). LAD1 is caused by defective leukocyte adhesion to endothelial cells and the absence of transmigration into inflamed tissues with deficient phagocytosis and chemotaxis (Arnaout and Michishita, 1993; Etzioni and Harlan, 2007). LAD1 patients have impaired cell surface expression of leukocyte adhesion molecules β2 integrin heterodimers: LFA-1 (CD11a/CD18), Mac-1 or CR3 (CD11b/CD18), p150/95 (CD11c/CD18) and CD11d/CD18 (Fagerholm et al., 2019; Bednarczyk et al., 2020). Abnormal β2 integrin expression is due to heterogeneous defects in the common β2 (CD18) subunit (Kishimoto et al., 1989; Matsuura et al., 1992; Rodríguez et al., 1993). The disease course can be severe (<2%) or moderate (2%–30%) (Anderson et al., 1985; Novoa et al., 2018). Multiple studies have revealed the molecular genetic heterogeneity underlying this disease. Several mutations scattered throughout the ITGB2 gene have been described, with some resulting in single amino acid changes, whereas small deletions lead to the generation of frame shifts, premature stop codons or splicing defects (Wardlaw et al., 1990; Fathallah et al., 2001; Roos et al., 2002; Parvaneh et al., 2010; Van De Vijver et al., 2012; Tipu et al., 2020). Conventional treatment of LAD1 often involves antibiotic-based therapy and/or prophylactic or adjunctive immunoglobulin treatment (Yamazaki-Nakashimada et al., 2015).

Currently, severe LAD1 patients with less than 2% CD18 expression are successfully treated with bone marrow and other hematopoietic stem cell transplantation (HSCT) therapies (Jamal et al., 1998; Elhasid and Rowe, 2010; Stepensky et al., 2015). Bone marrow transplantation therapy requires donors with preferably matched human leukocyte antigen (HLA), related, haploidentical, or unrelated HLA–matched hematopoietic stem cells. Despite the high rate of successful engraftment observed in patients with LAD1 and the limited although challenging graft-versus-host disease (GVHD), not all patients can undergo timely and completely safe HSCT (Bakhtiar et al., 2021). Indeed, inflammatory complications associated with the impaired IL-12/IL-23 pathway resulting in an IL-17–mediated inflammatory disease observed in LAD1 (Moutsopoulos et al., 2014) may occur during allo-HSCT, which requires anti-inflammatory therapy pretreatment. Gene therapy was developed for the treatment of primary immune deficiency (Kohn et al., 2021). Indeed, retroviral-mediated transfer to a lymphoblastoid B-cell line derived from an LAD patient (Back et al., 1990) and to a patient’s peripheral CD34^+^ cells (Bakhtiar et al., 2021) harboring a normal version of the ITGB2 gene rescued β2 integrin expression, indicating the potential of gene therapy for the treatment of LAD1. A clinical trial in which CD34+-enriched hematopoietic stem cells from subjects with severe LAD-I were transduced *ex vivo* with a lentiviral vector carrying the normal ITGB2 gene revealed the safety and efficacy of the procedure (https://clinicaltrials.gov/study/NCT03812263). Nevertheless, the recent advances in genome editing that allow changing an organism’s DNA at will and relatively easily seems to be a better alternative to HSCT and conventional vector-based gene therapy. Several approaches to genome editing have been developed (Gupta and Musunuru, 2014). A recent method is known as CRISPR-Cas9, which stands short for clustered regularly interspaced short palindromic repeats and CRISPR-associated protein 9. Compared with other genome editing methods, the CRISPR-Cas9 system is fast, inexpensive and more accurate and efficient. CRISPR-Cas9 consists of creating a small piece of RNA with a short “guide” sequence that attaches to a specific target sequence of DNA in the genome. This RNA is attached to the Cas9 enzyme. The modified RNA is used to recognize a defined DNA sequence, and the Cas9 enzyme cuts the DNA at this targeted location. Once the DNA is cut, the cell’s own DNA repair machinery is used to add or delete pieces of genetic material or to make changes to the DNA by replacing an existing segment with a customized DNA sequence (Hsu et al., 2014; Sander and Joung, 2014; Lander, 2016; Komor et al., 2017). Genome editing has been explored for the treatment of a wide variety of diseases, including single-gene disorders such as beta-thalassemia and sickle cell disease (Liang et al., 2017; Leonard et al., 2022), cystic fibrosis, and hemophilia (Rodríguez-Merchán et al., 2021; Ozelo et al., 2022; Wang, 2023). It has also been investigated for the treatment and prevention of more complex diseases, such as cancer, heart disease, neurodegenerative diseases, mental disorders and human immunodeficiency virus (HIV) infection (Lim, 2017; Rehman et al., 2020; Guan et al., 2022; Chehelgerdi et al., 2024; Kitawi et al., 2024).

We herein describe the use of *Jurkat* and patient-derived lymphoblastoid cell lines in the development of a CRISPR‒Cas9 protocol for the correction of a 10 bp deletion in two patients with the severe form of LAD1. We determined the right combination of gRNA and HDR to correct the genomic defect and reach a level of CD18 expression that reverses the phenotype of the patient’s lymphoblastoid-cell lines without recording any off-target modifications of the genome. Such protocol could be translated into a gene therapy protocol for LAD1 using the patients PMN cell progenitors.

## 2 Materials and methods

### 2.1 EBV cell lines

We used two lymphoblastoid cell lines (EBV-transformed B cells) previously established in our laboratory from two unrelated LAD one patients with the severe form of the disease (with no detectable expression of CD18 at the leukocyte cell surface) (Barbouche et al., 2017). The cells were cultured in RPMI-1640 medium (Gibco, United States of America) supplemented with 200 mM L-glutamine, 1% antibiotic antimycotic, and 15% fetal calf serum. The culture was maintained at 37°C in a 5% CO_2_ humidified incubator. Following three passages, the cells were collected, and a master cell bank was prepared.

### 2.2 Bioinformatics design of primers, gRNA and HDR

**FIGURE 1:**
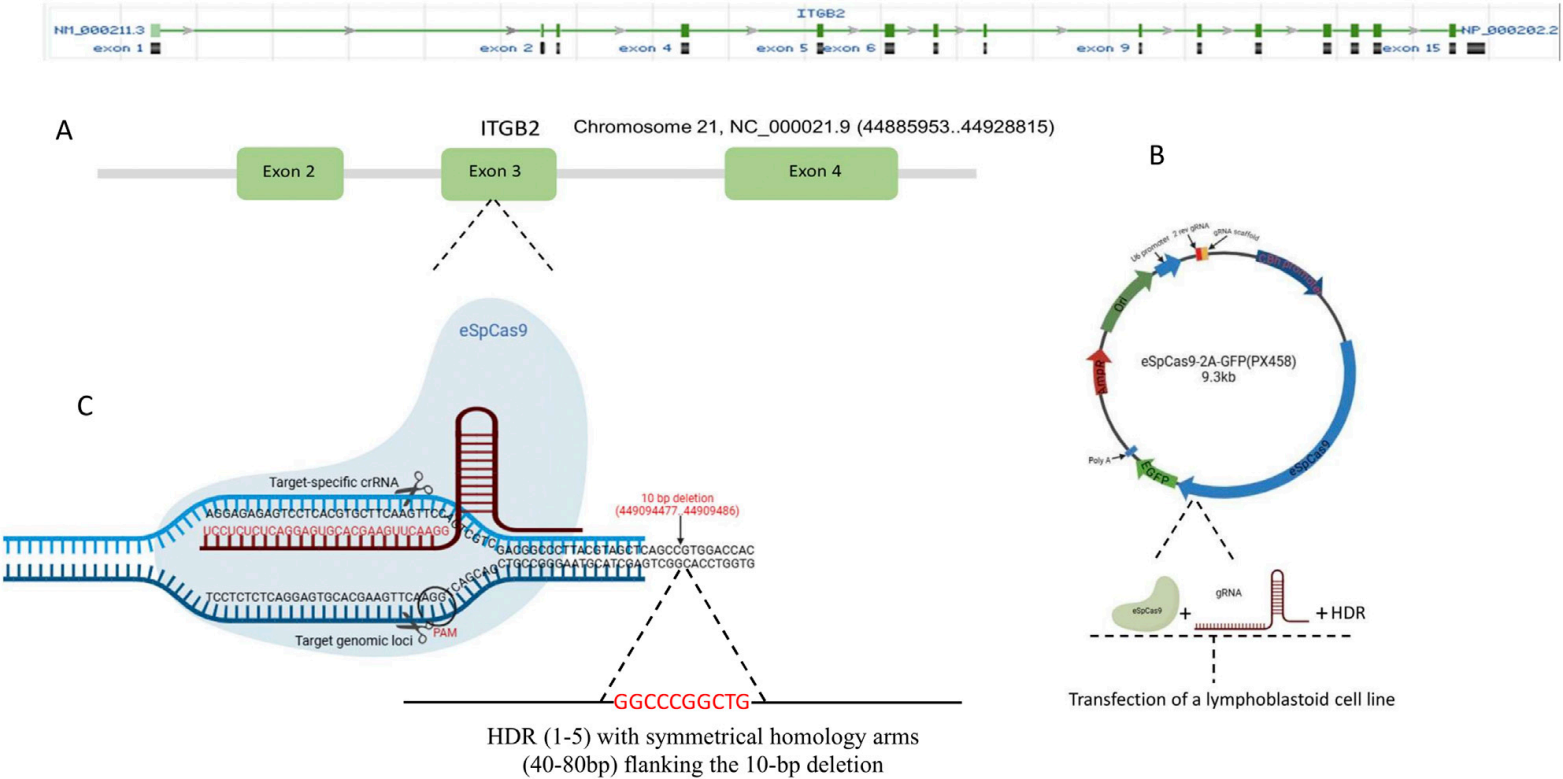
Strategy for correcting the 10-bp deletion in exon three of the *ITGB2* gene. This figure provides a schematic representation of the gene-editing strategy used to restore the *ITGB2* gene sequence **(A)** ITGB2 gene organization and target exon (exon 3) **(B)** The espCas9(1.1) plasmid was used for the expression of gRNAs and eSpCas9 in *Jurkat* and Lymphoblastoid Cell lines **(C)** The CRISPR-Cas9 gene-editing approach employed in this study is illustrated. Homology-directed repair (HDR) templates were designed as single-stranded donor oligonucleotides (ssODNs) with symmetrical homology arms flanking the 10-bp deletion. Five HDR templates, ranging in length from 90 to 170 bp, were used.

The RNA-guided endonuclease (RGEN) was designed via the CRISPOR website http://crispor.tefor.net/. Unique target sequences for the guide RNA were selected and optimized to avoid or reduce RGEN-induced off-target mutations. The Primer3 website https://primer3.ut.ee/was used to design specific primers and probes. HDR templates were designed as single-stranded donor oligonucleotides (ssODNs) with symmetrical homology arms flanking the 10-bp deletion. We designed five HDRs of lengths ranging from 90 to 170 bp. The HDR oligos were synthesized by Metabion (Germany) with HPLC purification to ensure high fidelity. espCas9(1.1) plasmid was used to express the gRNAs and Cas9 gene. The gene editing strategy employed for the study is depicted in Figure 1. Supplementary Table S1 lists all the primers, HDRs and gRNAs used.

### 2.3 PCR amplification and sequencing of the ITGB2 gene

To confirm the 10 bp deletion in the patients’ genomic DNA and check for DNA sequence repair following the CRISPR-Cas-9 protocol, we designed several primers that flank the 10 bp deletion (Supplementary Table S1) and yield an 881 bp PCR fragment. The PCR mixture consisted of 25 µL of 2x Qiagen master mix, 2 µL (10 pmol/μL) of forward primer, 2 µL (10 pmol/μL) of reverse primer, 2 µL of DNA, and 19 µL of nuclease-free water. The PCR cycle was as follows: 5 min at 95°C, followed by 35 cycles of denaturation at 95°C for 45 s, annealing at 60°C for 30 s, extension at 72°C for 1 min, and a final extension at 72°C for 5 min. The PCR products were sequenced on a 3500xL Genetic Analyzer (Applied Biosystems, United States) via the BigDye Terminator v3.1 Cycle Sequencing Kit (Applied Biosystems, United States). The results were analyzed via ABI Sequence Navigator software, version 3.1.

### 2.4 gRNA selection and optimization

To select and optimize the gRNA conditions and specific genomic DNA cleavage, *Jurkat* cells were used as a model. The cells were cultured in 12-well plates at a density of 2 × 10^5^ cells per well in 1 mL of complete RPMI-1640 medium containing 15% FBS (Gibco, United States). Transfection was conducted in accordance with the manufacturer’s instructions for Lipofectamine 3,000 and polyethyleneimine (PEI). Following a 72-hour incubation period, genomic DNA was extracted and subjected to genomic cleavage detection (GCD).

### 2.5 Genomic cleavage detection (GCD)

A GeneArt Genomic Cleavage Detection Kit (Thermo Fisher Scientific, United States) was used to measure the efficiency of genomic cleavage. The T7 endonuclease I-based method was utilized to quantify the editing protocol, which results in indels. The targeted locus was amplified from extracted DNA via PCR (Supplementary Table S1), and the cells were typically analyzed 72 h after transfection. The amplicon is denatured and reannealed, creating mismatched duplexes with strands containing indels annealed to strands without indels or strands with different indels. The detection enzyme cleaves the mismatched DNAs. The PCR product enzyme mixture was subsequently analyzed via agarose gel electrophoresis to determine the cleavage efficiency by comparing the relative proportions of cleaved and noncleaved bands in the gel. The gRNA with the highest editing efficiency near the insertion site was selected for subsequent homologous recombination assays.

### 2.6 Cell transfection (with selected gRNA and five HDRs)

The selected gRNA plasmids were used to transfect patients’ EBV cell lines with HDR single-stranded oligonucleotides. Polyethylenimine (PEI) was used for B lymphoid cell line transfection with the addition of HDR single-stranded oligonucleotides at 1 nmol per transfection. The cells were cultured for 24 h prior to transfection at a density of 3 × 10^6^ in a T25 cm flask. The cells were then transfected with 3 µg of CRISPR-Cas9 gRNA plasmid together with 18 µg of PEI (1 mg/mL) and 10 µL (1 nmol) of each HDR in separate transfections. One tube contained 3 µg of plasmid and 10 µL (1 nmol) of HDR diluted in 200 µL of RPMI-1640 Basel medium (Gibco, United States), while the second tube contained 18 µg of PEI (1 mg/mL) diluted in 200 µL of the same medium. The contents of tube one were then mixed with those of tube two for 15 min at room temperature, after which the resulting mixture was gradually added to the cells and incubated at 37°C for 5 h. At the end of this incubation period, 8 mL of complete RPMI-1640 medium (Gibco, United States) was added to the cells, which were subsequently incubated for an additional 72 h in a CO_2_ incubator. The cells were then used for DNA extraction and detection of gene editing and for flow cytometry to detect CD18 expression.

### 2.7 Detection of gene editing

#### 2.7.1 ARMS PCR

The reaction mixture included 7.5 µL of 2x master mix (Qiagen, Germany), 1 µL of forward primer (10 pmol/μL), I µl of reverse primer (10 pmol/μL) and 2 µL of extracted DNA, and the volume was increased to 15 µL via NFW. The PCR amplification consisted of initial denaturation for 5 min at 95°C followed by 35 cycles at 95°C for 1 min, 62°C for 30 s, and 72°C for 30 s and a final extension at 72°C for 10 min. The PCR products were subjected to 2% agarose gel electrophoresis.

#### 2.7.2 Real-time PCR

Wild type DNA was utilised as the positive control DNA, while patient DNA from unedited sample and mock-transfected sample was employed as the negative control DNA. An Applied Biosystems 7,500 instrument was used, and the reaction mixture included 7.5 µL of Taqman universal master mix (Applied Biosystems, UK), 1 µL of forward primer (10 pmol/μL), 1 µL of reverse primer (10 pmol/μL), 1 µL of probe, 2 µL of DNA and 2.5 µL of NFW. The PCR conditions were as follows: 2 min at 50°C and 10 min at 95°C, followed by 40 cycles at 95°C for 30 s and 1 min at 60°C for fluorescence detection. FAM channel used for fluorescence capture.

#### 2.7.3 Digital PCR detection

A QIAcuity One 5pLex Device (Qiagen, Germany) was used, and the reaction mixture was as follows: 10 µL of QIAcuity Probe PCR master mix (Qiagen, Germany), 2 µL of forward primer (10 pmol/μL), 2 µL of reverse primer (10 pmol/μL), 1 µL of probe, 2 µL of DNA (10 ng/μL) and 1 µL of EcoRI (12 units/μL) for DNA fragmentation to ensure the even distribution of the template throughout the QIAcuity Nanoplate. The final volume was 40 µL with nuclease-free water. Assembled reactions were transferred into QIAcuity 26k 24-well Nanoplates (Qiagen) for partitioning via the Qiagen Standard Priming Profile. PCR conditions were as follows: 2 min at 95°C, followed by 40 cycles at 95°C for 15 s and 30 s at 60°C for fluorescens collection. The positions were imaged with a 400 m (FAM) exposure time, with the gain set to six for the FAM channels. The QIAcuity Software Suite (Qiagen, version 2.1.7) was used to determine sample thresholds via positive, negative, and no-template control wells (NTCs) via the manual global threshold approach, which is based on the amplitude signal observed in the negative control sample.

### 2.8 Detection of CD18 expression via flow cytometry

Cells were centrifuged at 100 rpm and then washed three times with 1X PBS. Cell counting was performed via trypan blue dye and adjusted to 1 × 10^7^ cells/mL in PBS. For each culture, two tubes were run: one without an autofluorescence antibody and the second labeled with 5 µL of FITC-conjugated anti-human CD18 monoclonal antibody (200 μg/mL) (BioLegend, United States of America). After three washes, the cells were incubated on ice in the dark for 30 min, washed three times with 2 mL of PBS and centrifuged for 5 min at 1000 RPM. Finally, the cells were resuspended in 500 µL of PBS, and fluorescence was measured on a BD FACS Calibur Flow Cytometer (BD Biosciences, United States of America). The number of cells in each tube was 5 × 10^4^. Forward scatter (FSC) and side scatter (SSC) were adjusted to exclude debris and dead cells. The percentage of CD18 expression on the cell surface was calculated from positive FITC signals.

### 2.9 Whole-genome sequencing (WGS)

After gene editing of the LAD patient genome, the transfected cells were harvested, and genomic DNA was extracted via QIAamp DNA Blood Kits. The quality and quantity of the extracted DNA were assessed via a Qubit dsDNA HS Assay and agarose gel electrophoresis. For sequencing, library preparation was performed via the Twist Whole Genome Library Prep. After preparation, quality control (QC) analysis was conducted with a Qubit and BioAnalyzer to determine the concentration and fragment size distribution of the DNA. Successfully validated libraries were subjected to qPCR analysis via the KAPA library quantification kit on the QuantStudio system. Samples were pooled in equimolar or user-defined ratios to achieve the appropriate multiplexing level for the Illumina NovaSeq 6,000 platform, where sequencing was performed with paired-end 150 bp reads and an average coverage of 30X per sample.

#### 2.9.1 Bioinformatics analysis

The bioinformatics workflow started with the processing of raw WGS reads, ensuring high data quality. Quality control was first carried out via FastQC, and adapter trimming and low-quality base removal were performed via FastqMcf. The cleaned reads were aligned to the reference genome via BWA-MEM, and PCR duplicates were removed with Sentieon Dedup Picard to minimize biases. Next, indel realignment was performed with the Sentieon Realigner to correct misalignments near insertions and deletions, improving the accuracy of variant calling. The base quality scores were recalibrated via Sentieon QualCal to correct for systematic sequencing errors. Quality metrics were generated via Picard tools (CollectRawWgsMetrics and CollectWgsMetrics) to evaluate data integrity.

Variants were then identified with the Sentieon Haplotyper, and the initial set of SNP and indel variants was called via HaplotypeCaller in VCF mode. The raw variants were filtered such that only high-confidence SNPs and indels were retained. Variants were functionally annotated via VariMAT, which provided insights into their potential impact. Finally, to identify gene-editing-induced changes, VCF output files from CRISPR-Cas9 treated samples were compared against those from untreated control samples, allowing for the detection of specific variant profile differences and confirming the successful introduction of desired edits in target genes.

## 3 Results

### 3.1 Confirmation of LAD1 patient genetic defects

**FIGURE 2:**
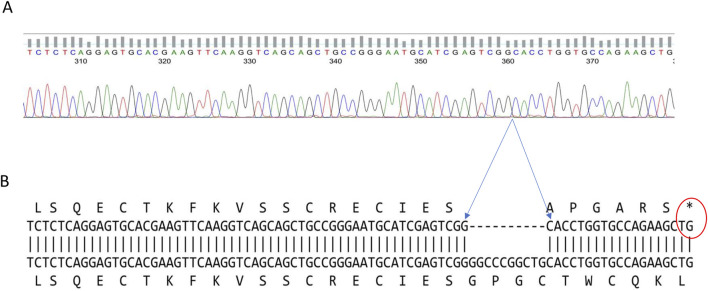
Confirmation of the LAD1 patient’s ITGB2 gene defect. The two patients’ DNA sequence have a 10-base pair deletion at exon three of the ITGB2 gene **(A)** Display of the DNA sequence generated using the forward primer **(B)** Amino acid alignment of the patient sequence versus reference genome shows the position of the stop codon generated by the 10bp deletion that caused the frameshift, and the premature termination (Gly40AlafsX7).

To confirm the presence of the 10-bp deletion associated with Leukocyte Adhesion Deficiency Type 1 (LAD1), we analyzed the ITGB2 gene in patient-derived EBV-transformed B-cell lines. The patients’ homozygous del (GGCCCGGCTG) mutation at position 23,524 of the ITGB2 gene was identified through PCR amplification and Sanger sequencing. The resulting amplicon sequences were aligned against the reference *Homo sapiens* integrin subunit beta-2 (ITGB2) sequence (RefSeqGene LRG_76, Sequence ID: NG_007270.2) from the NCBI Data Bank to confirm the precise deletion (Figure 2A).

Further *in silico* translation analysis revealed that this deletion, located in exon three of the ITGB2 gene, results in the loss of amino acid residues 41–43. This leads to a frameshift mutation and the introduction of a premature termination codon (Gly40Alafs17), occurring 17 base pairs downstream of the deleted region (Figure 2B).

### 3.2 *In silico* preselection of gRNAs

To identify optimal guide RNA (gRNA) sequences for CRISPR-Cas9 mediated correction of the 10-bp deletion in ITGB2, we analyzed the surrounding DNA sequence using the CRISPOR web tool (Haeussler et al., 2016). This analysis generated twelve potential gRNAs (Supplementary Table S2), ranked based on predicted on-target efficiency and specificity scores.

From this set, three gRNAs were preselected based on their Doench ‘16 efficiency score, MIT specificity score, and CFD score, which assess cleavage potential and minimize off-target effects (Table 1). The PAM sites and expected cleavage positions were also considered, with cleavage predicted to occur three base pairs upstream (5’) of the PAM sequence. These preselected gRNAs were further tested experimentally to determine the most efficient gRNA-HDR combination for gene correction. The selection criteria for gRNA ranking were as follows: Doench ‘16 efficiency score (Doench et al., 2016) which predicts the likelihood of strong versus weak cleavage, with higher scores correlating with increased efficiency, MIT specificity score which ranges from 0 to 100, summarizing potential off-target interactions; higher scores indicate fewer off-target effects and finally, the CFD score which represents the Cas9-target interaction strength, also ranging from 0 to 100. A score of 100 indicates optimal guide-target binding, while lower values suggest mismatches that may reduce editing precision.

**TABLE 1:** Selected gRNAs.

gRNA					
Position/Strand	Guide sequence and PAM	MIT Specificity Score	CFD Specificity score	Off-targets for 0-1-2-3-4 mismatches	Predicted Efficiency Doench ‘16-Score
2rev	ACT​TCG​TGC​ACT​CCT​GAG​AG AGG	77	90	0 - 0 - 1 - 4 - 83 0 - 0 - 1 - 2 - 2 88 off-targets	64
28forw	TCA​GGA​GTG​CAC​GAA​GTT​CA AGG	87	91	0 - 0 - 1 - 9 - 83 0 - 0 - 0 - 0 - 0 93 off-targets	47
41rev	GCC​CCG​ACT​CGA​TGC​ATT​CC CGG	96	98	0 - 0 - 0 - 0 - 26 0 - 0 - 0 - 0 - 0 26 off-targets	39

### 3.3 Experimental selection of gRNAs

**FIGURE 3:**
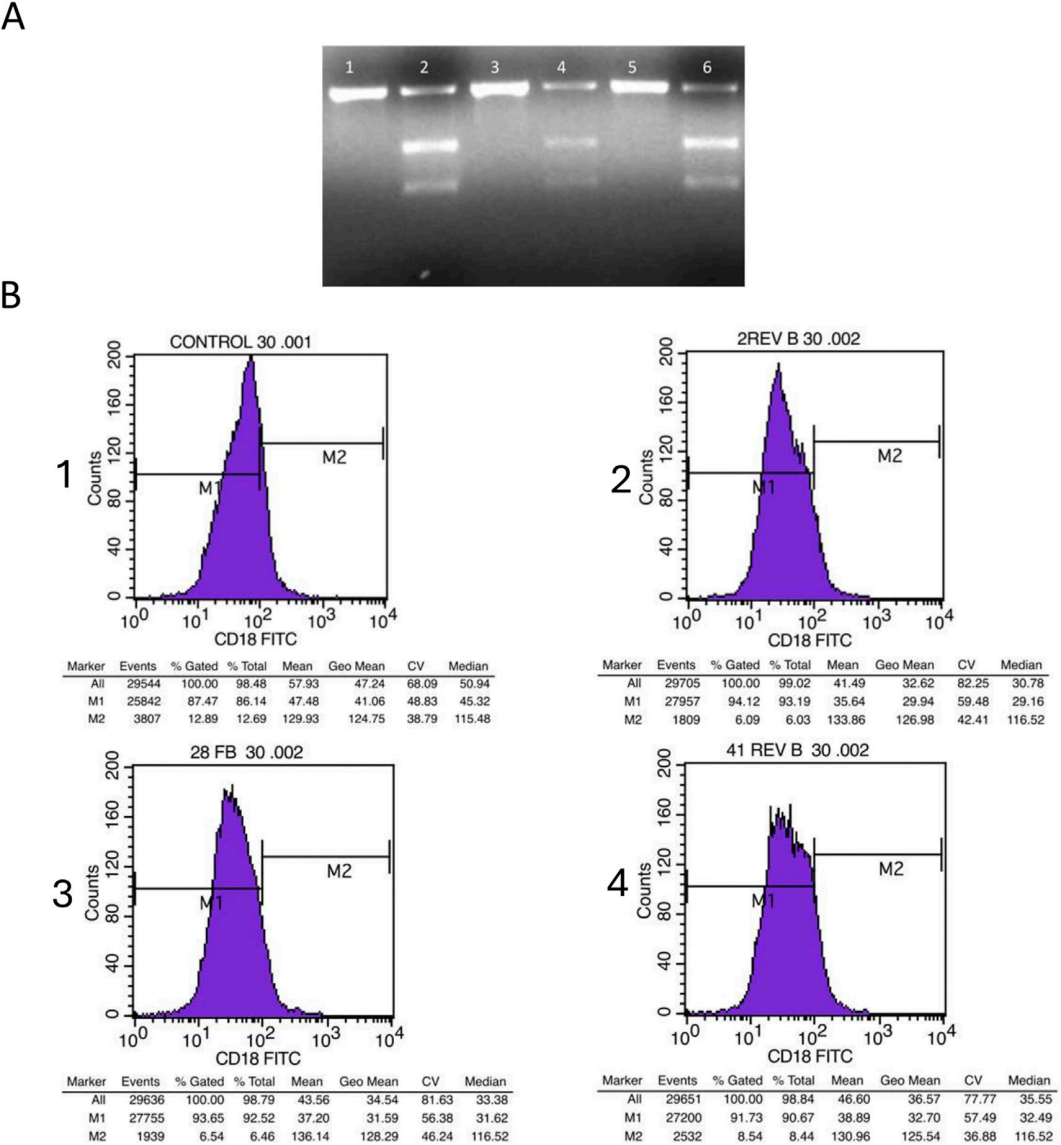
Analysis of *Jurkat* cells Genomic cleavage and CD18 expression following transfection with gRNAs **(A)** Agarose gel electrophoresis of the amplicon covering the gRNA Cas9 specific cut site, after 72 h of transfection. Lane one displays the uncut PCR for cells that were transfected with 2-rev gRNA, while lane two displays cut GCD PCR for cells that were transfected with 2-rev gRNA. Lane three displays uncut PCR for cells transfected with 28-for gRNA, while lane four displays cut GCD PCR for cells transfected with 28-for gRNA. Lane five displays uncut PCR for cells transfected with 41-rev gRNA, and lane six displays cut GCD PCR for cells that were transfected with 41-rev gRNA **(B)** Flow cytometry histograms illustrating the level of CD18 *Jurkat* cell surface expression transfected with the three gRNA. CD18 expression on non-transfected cells (1). CD18 expression on cell transfected with 2-rev gRNA (2). CD18 expression on cell transfected with 28-for gRNA (3). CD18 expression on cell transfected with 41-rev gRNA (4).

The optimal guide RNA (gRNA) was selected based on its genomic DNA cleavage efficiency and the subsequent reduction in CD18 surface expression in *Jurkat* cells. The three preselected gRNAs were tested for their ability to induce genomic cleavage and disrupt ITGB2 expression following transfection. Genomic cleavage was assessed 72 h post-transfection using the GeneArt Genomic Cleavage Detection (GCD) Kit, while CD18 expression levels were measured via flow cytometry. All three gRNAs successfully induced DNA cleavage; however, as shown in Figure 3A, the 2-rev gRNA demonstrated the highest cleavage efficiency. Figure 3B illustrates the flow cytometry analysis of CD18 expression on the surface of *Jurkat* cells transfected with each gRNA construct. The results indicate that the 2-rev gRNA caused the most significant reduction in CD18 expression, as further quantified in Table 2, which presents the percentage of CD18-positive cells following transfection with each gRNA. The combined *in silico* preselection and experimental validation confirmed that 2-rev gRNA exhibited the highest editing efficiency. Based on these results, the 2-rev gRNA plasmid was selected to evaluate five different HDR templates for genomic repair in patient-derived EBV cell lines.

**TABLE 2:** Evaluation of the ITGB2 gene knock out in *Jurkat* cells. The reduction of the CD18 expression levels on the surface of *Jurkat* cells transfected with the three selected gRNAs reflects the percentage of the cells where the ITGB2 gene was knocked out.

Cells	Total of event	Positive CD18 expression on cell surface	% Reduction in cell surface CD18 expression
Control	3 × 10^4^	12.89	
2-rev	3 × 10^4^	6.09	52.8%
28-forw	3 × 10^4^	6.54	49.3%
41-rev	3 × 10^4^	8.54	33.38%

### 3.4 Correction of LAD1 patients’ genomic defects

Following the selection of the gRNA (2-rev gRNA), each patient’s EBV cell line was co-transfected with the 2-rev gRNA and each of five different HDRs. These HDRs have the targeted 10 bp insertion sequence located at the center, flanked by 40, 50, 60, 70, and 80 bp on each side. The total lengths of the HDRs were 90, 110, 130, 150, and 170 bp, respectively. For the negative control, patient EBV cell lines were transfected with a mock CRISPR-Cas9 plasmid. The genomic DNA was extracted 72 h post transfection, and gene repair was detected via four different molecular methods. Additionally, the cells were analyzed by flow cytometry to determine the CD18 expression level on the cell surface.

#### 3.4.1 DNA sequencing

**FIGURE 4:**
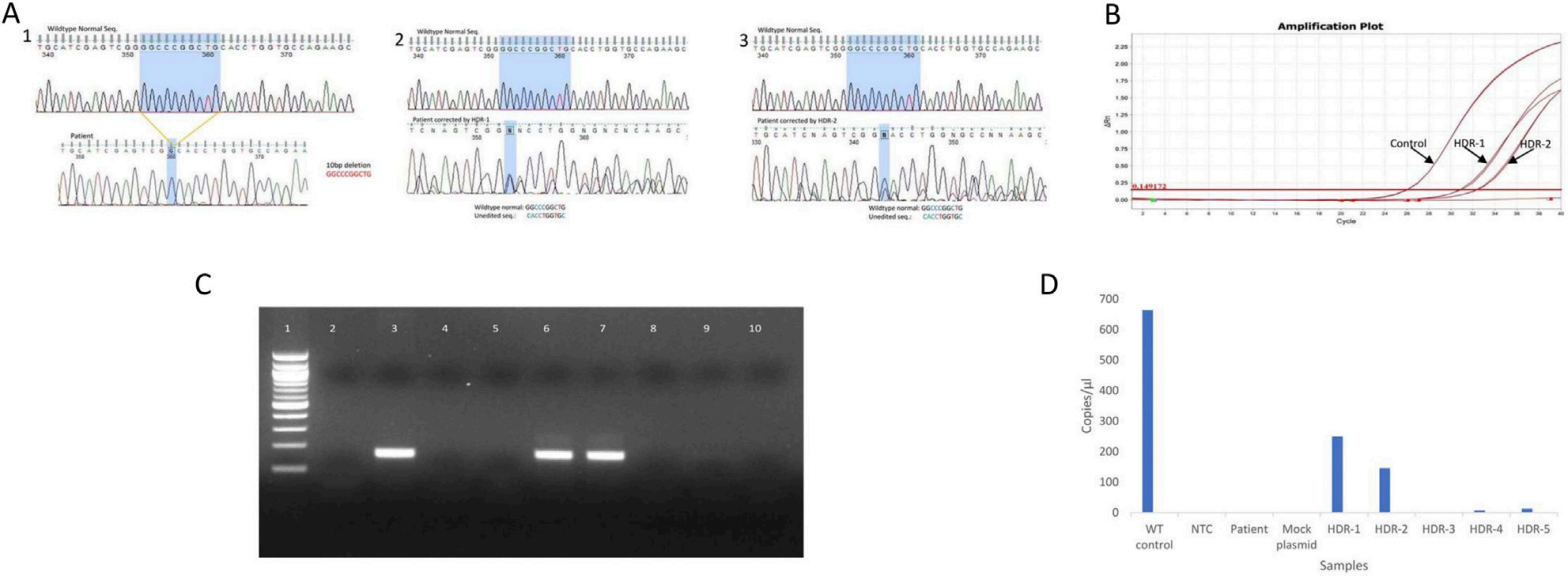
Confirmation of LAD1 patient 10 bp genomic correction by various molecular methods: Direct PCR-Sequencing, Real time PCR, ARMS PCR and digital PCR **(A)** DNA sequencing electrogram of wild type sequence and 10bp deletion (1), DNA sequence corrected by HDR-1 (2), DNA sequence corrected by HDR-2 (3). The shaded areas correspond to the deleted 10bp **(B)** Real time amplification plot for edited gene using probe cover 10bp deletion, showing the liner change of the fluorescence plotted versus cycle number. The horizontal read line indicates the threshold value of fluorescence. Control: indicate amplification of normal sample, HDR-1: indicate amplification of sample corrected with HDR-1, HDR-2: indicate amplification of sample corrected with HDR-2 **(C)** Agarose gel electrophoresis representing the ARMS PCR for cells transfected with mock plasmid and different HDR length. Lane1: 100 bp DNA ladder, lane2: NTC, lane3: wild type control, lane4: PCR from un-corrected cells, lane 5: PCR from cells transfected with mock plasmid, lane 6: PCR from cells transfected with HDR-1, lane 7: PCR from cells transfected with HDR-2, lane 8: PCR from cells transfected with HDR-3, lane 9: PCR from cells transfected with HDR-4, lane 10: PCR from cells transfected with HDR-5 **(D)** Digital PCR concentration (copies/μL) of CD-18. Wild-type control (664 copies/μL), no template control (1 copy/μL), patient (1 copy/μL), mock plasmid: patient transfected with mock plasmid (1 copy/μL), HDR-1: patient transfected with 2-rev plasmid and HDR-1 (250 copies/μL), HDR-2: patient transfected with 2-rev plasmid and HDR-2 (146 copies/μL), HDR-3: patient transfected with 2-rev plasmid and HDR-3 (0 copy/μL), HDR-4: patient transfected with 2-rev plasmid and HDR-4 (7 copies/μL), HDR-5: patient transfected with 2-rev plasmid and HDR-5 (13 copies/μL).

The DNA sequencing of the PCR product covering the genomic alteration revealed that the 10-base pair deletion was corrected in the cells that were transfected with the 2-rev plasmid and HDR-1 or HDR-2. The electropherogram of the sequence (Figure 4A) shows heterozygosity due to the presence of a mixed population of corrected and uncorrected sequences.

#### 3.4.2 Real-time PCR detection of gene editing

To evaluate the correction of the 10 bp deletion by real-time PCR, we used a probe that covers the area targeted for repair. Following cell transfection with gRNA and HDRs, samples were collected after 72 h and analyzed via real-time PCR. Figure 4B shows amplification in the sample corrected with HDR-1 and HDR-2 in comparison with the control sample, but no amplification was detected in HDR-3, HDR-4, HDR-5, the mock plasmid, or the patient sample prior to correction.

#### 3.4.3 ARMS PCR detection of gene editing

The reverse ARMS primer was designed to anneal with the 3′ end of the missing 10 bp. If the DNA is corrected, the target sequence is amplified, resulting in a PCR product of a specific size. If not, no PCR product can be observed. As shown in Figure 4C, strong amplification occurred in samples transfected with HDR-1 and HDR-2. The samples transfected with HDR-3, HDR-4, or HDR-5 exhibited very weak amplification. The sample transfected with the mock plasmid showed no amplification.

#### 3.4.4 Digital PCR detection of gene editing

Digital PCR was carried out with genomic DNA from unedited patients, EBV cells transfected with a simple Cas9 plasmid (mock), cells transfected with different HDRs and a wild-type positive control DNA along with a non-template control (NTC). The QIAcuity One 5pLex device detected the concentration of corrected DNA in copies per microliter via the 2-Rev plasmid and the five HDRs. Figure 4D presents the concentration of positive partitions The concentrations of CD18 were 664, 250 and 146 copies/μL for the Wilde-type control, patient-transfected with the 2-rev plasmid and HDR-1, and patient-transfected with the 2-rev plasmid and HDR-2, respectively.

#### 3.4.5 Detection of the CD18 receptor on edited cells via FACS analysis

**FIGURE 5:**
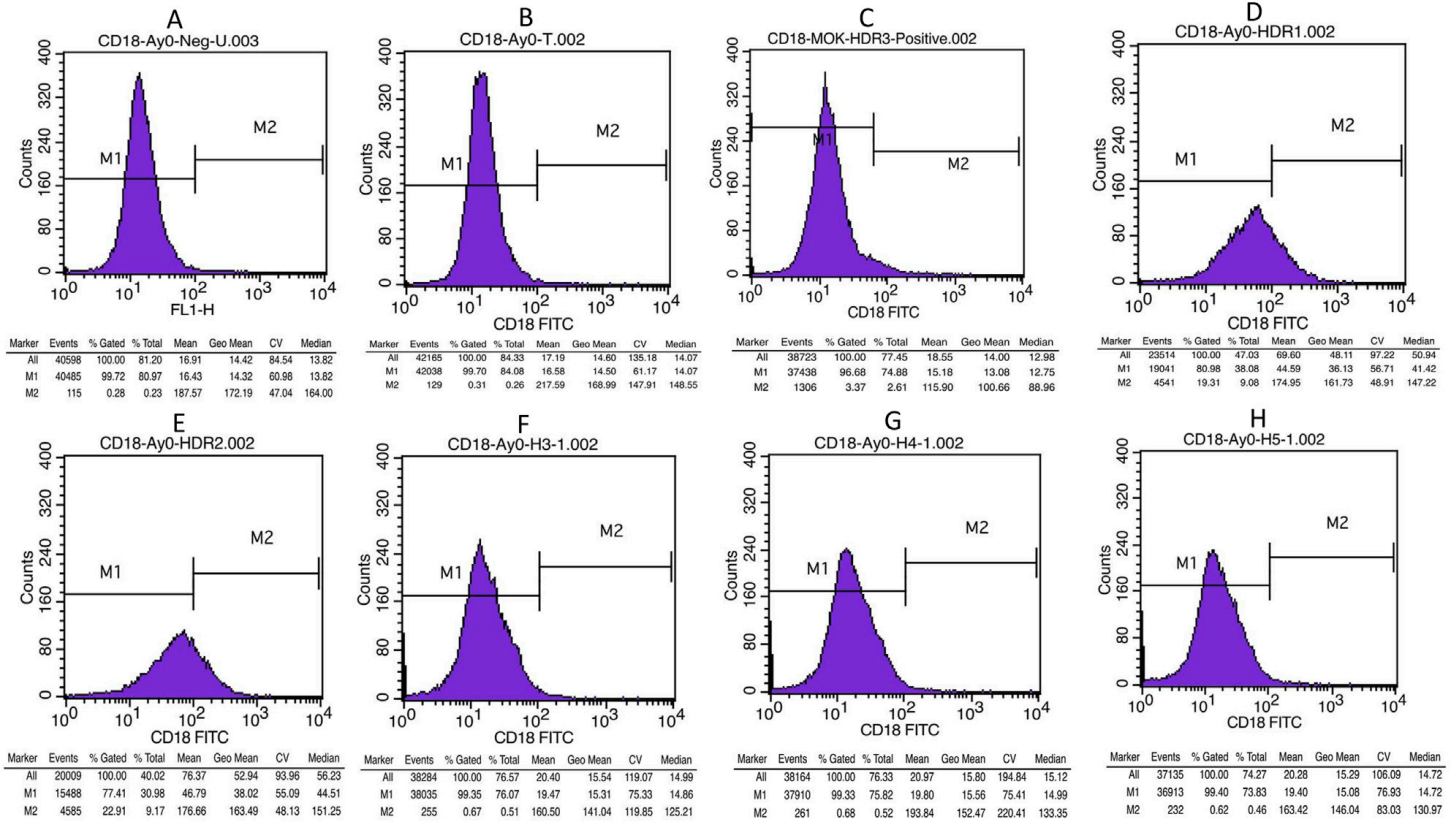
Flow cytometry histogram results shows CD18 expression in controls and edited cells **(A)** Unstained control **(B)** Un-transfected patient cells, **(C)** Cells transfected with mock plasmid **(D)** Cells transfected with 2-rev plasmid and HDR-1, **(E)** Cells transfected with 2-rev plasmid and HDR-2 **(F)** Cells transfected with 2-rev plasmid and HDR-3, **(G)** Cells transfected with 2-rev plasmid and HDR-4 **(H)** Cells transfected with 2-rev plasmid and HDR-5.

Prior to gene editing, the patient exhibited no expression of CD18 on the surfaces of their cells due to frameshift and stop codon mutations. To assess the effectiveness of gene editing, we measured the expression of CD18 receptors on the surface of patients’ edited EBV cells via flow cytometry with a CD18-specific monoclonal antibody. A total of 50,000 cells were analyzed for CD18 expression. No or very low expression (<1%) was detected in unstained control cells, non-transfected cells, cells transfected with mock plasmid, and cells transfected with HDR-3, HDR-4 or HDR-5. However, cells transfected with HDR-1 and cells transfected with HDR-2 presented expression levels of 19% and 23%, respectively, as shown in Figure 5.

### 3.5 Confirmation of genomic defect repair and identification of eventual off-target genomic events via whole-genome sequencing

Whole-genome sequencing of the unedited control sample and a sample edited with 2-rev gRNA and HDR-1 revealed the following key findings: a 10 bp deletion (GGCCCGGCTG) on chromosome 21:44910302 was detected in the control sample but was absent in the edited sample, even under low coverage, indicating successful correction at this site. The CRISPR web tool (CRISPOR) was used to predict 82 off-target sites for the 2-rev gRNA. All the predicted off-target sites were examined, and no genomic changes were recorded in the edited sample, as shown in Supplementary Table S3. Long insertion sequences longer than 50 bp were subjected to a BLAST search of the NCBI database, and none exhibited homology to the HDR-1 sequence.

These results demonstrate precise and efficient gene editing with presumably no unintended off-target effects or long insertions related to HDR-1.

## 4 Discussion

Inborn errors of immunity embody a set of serious life-threatening pathological conditions that can be reversed by gene therapy (Kohn et al., 2021). Nevertheless, gene therapy procedures are not yet widespread in the treatment of these difficult-to-cure diseases (Baylot et al., 2024). For LAD1 patients, hematopoietic stem cell transplantation is currently the main treatment for the severe form of the disease despite the serious health complications it may cause. Indeed, this approach is still considered a risky procedure limited by transplant-related toxicity and graft-versus-host disease. Interestingly, Moutsopoulos and his collaborators (Moutsopoulos et al., 2017) reported the successful treatment of an LAD1 patient with ustekinumab, an antibody that blocks signaling via the cytokines interleukin-23 and interleukin-12, inhibits IL17 and alleviates inflammatory lesions. Following treatment, the patient’s infections resolved, and infection-induced tissue damage was reduced. The levels of inflammation and serious infections decreased significantly without adverse reactions. Nevertheless, despite the observed restoration of impaired neutrophil function, this interesting immunotherapy approach remains a time point treatment because it does not restore β2 integrin expression and full functions. It is not a cure for LAD1. Therefore, gene therapy remains the best option for reversing this disease because its molecular root can be tackled by correcting genetic defects.

The first LAD1 gene therapy protocols relied mainly on supplying a wild-type version of the defective gene via a retroviral vector, including a self-inactivated lentivirus. Despite the initial reports of safety and efficacy, the procedure remains challenging and still requires myeloablative conditioning (Bauer Jr et al., 1998; Kohn et al., 2021). The recent developments in accurate and versatile (easy and robust) gene editing technologies heralded a new era of precise and more controlled gene therapy protocols. LAD1 is essentially caused by small defects in the ITGB2 gene and is thus prone to genetic correction via gene editing tools, particularly CRISPR-Cas9. The success of CRISPR-Cas9 in correcting small genomic defects depends upon using the right gRNA/HDR combination that do not cause off target genomic events. In this work, we investigated the use of *Jurkat* and lymphoblastoid cell lines in developing a CRISPR-Cas9 gene editing method. To develop this method, we used *Jurkat* cell line to determine which gRNA is the most efficient for specific genomic DNA cleavage while we used Lymphoblastoid cell lines established from the patient to determine the best gRNA and HDR combination. Indeed, *Jurkat* and the lymphoblastoid-cell lines allow multiple experiments to define the best conditions for correcting a genomic defect without resorting to the patient’s cells, particularly progenitor cells, which are not abundant enough to carry out the experiments needed to develop the optimal CRISPR-Cas9 method. This method circumvents the use of CD34^+^ progenitor cells while allowing the determination of the best gRNA and HDR for optimal CRISPR-cas9 gene editing. Yet, clinical application of our method will require validation in CD34^+^ cells to ensure safety, efficacy, and long-term stability. In this method, the transfection of lymphoblastoid cell line (B-LCLs) is challenging, and optimization is needed (Johnston et al., 2019). In this CRISPR method, we tested the concentrations of PEI, three CRISPR-Cas9/gRNAs and five HDRs at different concentrations along with different cell densities at the time of transfection. To improve LCL cell transfection, further optimization can be carried out via an experimental design.

For the experimental testing of the three predicted gRNAs, we used *Jurkat* cells as a prototypical cell line to determine which gRNA is the most efficient for specific genomic DNA cleavage. This step cannot be carried out using a patient’s lymphoblastoid cell line whose genome carries a genetic defect causing deficiency in integrin CD11/CD18 expression. The *Jurkat* cell line is an immortalized T lymphocyte cell line that expresses CD11/CD18 at its surface (Schneider et al., 1977) thus, it is suitable for testing gRNA genomic cleavage. On the other hand, our study did not include flow cytometry cell sorting of transfected LCLs because, ultimately, for the clinical treatment of LAD1 gene therapy, the genomic DNA of patient progenitor cells needs to be edited. Our focus was on developing a CRISPR-Cas9 protocol with optimized gRNA and single-stranded donor oligonucleotide or HDR to correct the specific 10 bp deletion. To evaluate the efficiency of HDR-mediated recombination, we analyzed the corrected sequences in gene-edited cells. Whole-genome sequencing confirmed the precise insertion of the 10-bp sequence without additional modifications at the Cas9 target site. Furthermore, no evidence of repeated cleavage or unintended indels at the corrected locus was detected, suggesting efficient HDR repair with minimal interference from non-homologous end joining (NHEJ). Indeed, the protocol we developed allowed a level of CD18 expression (19%–23%) on the patients’ B-cell surface that is sufficient to alleviate the clinical symptoms of LAD1 patients (Anderson et al., 1985; Novoa et al., 2018). However, despite the demonstration of successful correction of the ITGB2 gene defect and restoration of the CD18 expression by cytofluorometry, functional validation is required to assess the ability of edited cells to adhere to endothelial cells, migrate properly and carry out immune functions. This is particularly mandatory if the method we developed to determine the gRNA and HDR that give scareless genome editing is translated into a clinical gene therapy protocol using CD34^+^ hematopoietic stem/progenitor cells.

In addition, to develop this CRISPR‒Cas9 protocol, we used high-fidelity espCas9 to minimize off-target events (Cho et al., 2014). Indeed, espCas9 does not cleave genomic DNA if there are mismatches between the gRNA and the protospacer when random binding occurs (Ran et al., 2013; Fu et al., 2014; Slaymaker et al., 2016). Whole sequencing of the corrected genome did not reveal any off-target events, which proves the potential of using high-performance Cas9 protein in gene therapy via CRISPR in clinical settings. The success of this CRISPR gene editing method in correcting the genotype and reversing the phenotype of patients’ lymphoblastoid cell lines highlights the usefulness of LCLs as a model system for developing CRISPR–Cas9 based gene editing protocols. Indeed, it facilitates the identification of the best gRNA/HDR combination for an efficient genome editing that can be translated into a clinical gene therapy protocol for genetic diseases (Mittal, 2019).

In conclusion, this work shows that using a prototypical cell line such as *Jurkat* and the patient lymphoblastoid cell line as surrogates allow the development of a CRISPR-Cas9 method without having recourse to the patients’ hematopoietic stem cells or CD34^+^ peripheral blood cells. The determination of the best gRNA/HDR combination to correct genomic defects is essential for establishing an *in vivo* gene therapy procedure that can be optimized via the use of more advanced Cas9 (Pickar-Oliver and Gersbach, 2019). This method can be expanded to other LAD1 patients with various ITGB2 genomic defects to further assess the gene editing outcome across different genetic backgrounds and ensure its broader applicability.

## Data Availability

The datasets presented in this study can be found in online repositories. The names of the repository/repositories and accession number(s) can be found in the article/Supplementary Material.
