# Supplementary material for: Lymphoblastoid and Jurkat cell lines are useful surrogate in developing a CRISPR-Cas9 method to correct leukocyte adhesion deficiency genomic defect
Source: Front Bioeng Biotechnol. 2025 Mar 21;13:1548227. doi: 10.3389/fbioe.2025.1548227 (PMC11968696; doi:10.3389/fbioe.2025.1548227)
Supplement: Supplementary file 1 [file Table1.docx]

**Supplementary table 1**: Sequences of the different primers and HDRs (ssODN).

| Primers | | Sequence | Amplicon size bp |
| --- | --- | --- | --- |
| ARMS-PCR | Forward | 5-TCCTCTGACCTTCCTGCTCT-3 | 164 |
|  | Reverse | 5-CACCAGGTGCAGCCGGGCCC-3 |  |
| GCD | Forward | 5-TGTAGCTGTGCTGTTTCCCGAG-3 | 606 |
|  | Reverse | 5-CGTGTTTCTGCACTTGCTTACG-3 |  |
| RT-PCR/  D-PCR | Forward | 5-CAGGAGTGCACGAAGTTCAA-3 | 150 |
|  | Reverse | 5-GCTTCAGGGGCAGGAAAG-3 |  |
|  | Probe | FAM-AGTCGGGGCCCGGCTGCAC-BHQ 1* |  |
| Sequencing | Forward | 5-ACGCGTGACATTCCAATCAG-3 | 881 |
|  | Reverse | 5-ACATTTCCTCTTGGCCACCT-3 |  |
| HDR | | | |
| HDR-1 | CAGCCGGCCTCGTCCCTCAGTCCTCTCTCAGGAGTGCACGAAGTTCAAGGTCAGCAGCTGCCGGGAATGCATCGAGTCGGGGCCCGGCTGCACCTGGTGCCAGAAGCTGGTAAGTGCCTCCTGGACCCCTCCCCACCTGCCCAGCTCCTGGGTGAGGGCCCTTTCCTGCC | | 170 |
| HDR-2 | CGTCCCTCAGTCCTCTCTCAGGAGTGCACGAAGTTCAAGGTCAGCAGCTGCCGGGAATGCATCGAGTCGGGGCCCGGCTGCACCTGGTGCCAGAAGCTGGTAAGTGCCTCCTGGACCCCTCCCCACCTGCCCAGCTCCTGGGTGAGGGCC | | 150 |
| HDR-3 | TCCTCTCTCAGGAGTGCACGAAGTTCAAGGTCAGCAGCTGCCGGGAATGCATCGAGTCGGGGCCCGGCTGCACCTGGTGCCAGAAGCTGGTAAGTGCCTCCTGGACCCCTCCCCACCTGCCCAGCTCCTG | | 130 |
| HDR-4 | GGAGTGCACGAAGTTCAAGGTCAGCAGCTGCCGGGAATGCATCGAGTCGGGGCCCGGCTGCACCTGGTGCCAGAAGCTGGTAAGTGCCTCCTGGACCCCTCCCCACCTGC | | 110 |
| HDR-5 | AAGTTCAAGGTCAGCAGCTGCCGGGAATGCATCGAGTCGGGGCCCGGCTGCACCTGGTGCCAGAAGCTGGTAAGTGCCTCCTGGACCCCT | | 90 |

ARMS: Amplification-refractory mutation system, GCD: Genomic Cleavage Detection, RT-PCR: Real time PCR, D-PCR: Digital PCR, FAM: Fluorescein amidites, BHQ1*: Black Hole Quencher 1

**Supplementary table2:** Potential guide RNA sequences predicted by the CRISPOR website.

http://crispor.tefor.net/

| ^Position/^  ^Strand^ | ^Guide Sequence + PAM^ | ^MIT^  ^Specificity^ ^score^ | ^CFD pec. score^ | ^Off-targets for^  ^0-1-2-3-4 mismatches^ | ^Predicted Efficiency^  ^Doench '16-Score^ |
| --- | --- | --- | --- | --- | --- |
| ^60forw^ | ^GCCGGGAATGCATCGAGTCGGGG^ | ^98^ | ^99^ | ^0 - 0 - 0 - 1 - 23^  ^0 - 0 - 0 - 0 - 0^  ^24 off-targets^ | ^59^ |
| ^59forw^ | ^TGCCGGGAATGCATCGAGTCGGG^ | ^97^ | ^99^ | ^0 - 0 - 0 - 1 - 27^  ^0 - 0 - 0 - 0 - 0^  ^28 off-targets^ | ^39^ |
| ^41rev^ | ^GCCCCGACTCGATGCATTCCCGG^ | ^96^ | ^98^ | ^0 - 0 - 0 - 0 - 26^  ^0 - 0 - 0 - 0 - 0^  ^26 off-targets^ | ^39^ |
| ^58forw^ | ^CTGCCGGGAATGCATCGAGTCGG^ | ^95^ | ^97^ | ^0 - 0 - 0 - 0 - 40^  ^0 - 0 - 0 - 0 - 0^  ^40 off-targets^ | ^55^ |
| ^65forw^ | ^GAATGCATCGAGTCGGGGCCCGG^ | ^91^ | ^95^ | ^0 - 0 - 0 - 3 - 47^  ^0 - 0 - 0 - 0 - 1^  ^50 off-targets^ | ^41^ |
| ^28forw^ | ^TCAGGAGTGCACGAAGTTCAAGG^ | ^87^ | ^91^ | ^0 - 0 - 1 - 9 - 83^  ^0 - 0 - 0 - 0 - 0^  ^93 off-targets^ | ^47^ |
| ^2rev^ | ^ACTTCGTGCACTCCTGAGAGAGG^ | ^77^ | ^90^ | ^0 - 0 - 1 - 4 - 83^  ^0 - 0 - 1 - 2 - 2^  ^88 off-targets^ | ^64^ |
| ^75forw^ | ^AGTCGGGGCCCGGCTGCACCTGG^ | ^67^ | ^88^ | ^0 - 0 - 0 - 23 - 124^  ^0 - 0 - 0 - 3 - 5^  ^147 off-targets^ | ^45^ |
| ^63rev^ | ^TTCTGGCACCAGGTGCAGCCGGG^ | ^62^ | ^79^ | ^0 - 0 - 3 - 30 - 206^  ^0 - 0 - 1 - 1 - 7^  ^239 off-targets^ | ^49^ |
| ^42forw^ | ^AGTTCAAGGTCAGCAGCTGCCGG^ | ^53^ | ^80^ | ^0 - 0 - 3 - 25 - 232^  ^0 - 0 - 1 - 5 - 5^  ^260 off-targets^ | ^48^ |
| ^43forw^ | ^GTTCAAGGTCAGCAGCTGCCGGG^ | ^53^ | ^78^ | ^0 - 0 - 3 - 41 - 179^  ^0 - 0 - 1 - 7 - 16^  ^223 off-targets^ | ^53^ |
| ^64rev^ | ^CTTCTGGCACCAGGTGCAGCCGG^ | ^47^ | ^70^ | ^0 - 1 - 0 - 45 - 347^  ^0 - 0 - 0 - 2 - 2^  ^393 off-targets^ | ^41^ |

**Supplementary table3:** 2-rev gRNA predicted off-targets.

| Predicted off-targets | Chromosome | Number of mismatches ! |  |
| --- | --- | --- | --- |
| Exon:RP11-113I24.1 | chr1:189959924-189959946 | 4 | Absence |
| Intergenic:ACTA1-RP5-1068B5.3 | chr1:229,575,626-229,575,648 | 4 | Absence |
| Exon:IPO9 | chr1:201845526-201845548 | 4 | Absence |
| Intergenic:RP11-92G12.3-CAMSAP2 | chr1:200707787-200707809 | 4 | Absence |
| Intron:TRIM62 | chr1:33637970-33637992 | 4 | Absence |
| Intron:TOR3A | chr1:179055163-179055185 | 4 | Absence |
| Intergenic:PREB-C2orf53 | chr2:27358745-27358767 | 2 | Absence |
| Intron:AC114808.3 | chr2:1057939-1057961 | 4 | Absence |
| Intergenic:LTBP1-RNA5SP92 | chr2:33557907-33557929 | 4 | Absence |
| Intergenic:AC018890.6-CHRNA1 | chr2:175595452-175595474 | 4 | Absence |
| Intergenic:AC107079.1-AC112715.2 | chr2:238065877-238065899: | 4 | Absence |
| Intergenic:KLHL6-AS1-KLHL6 | chr3:183,271,315-183,271,337 | 3 | Absence |
| Intron:EPHB1 | chr3:134839988-134840010 | 4 | Absence |
| Intergenic:LINC00488-RNU6-1236P | chr3:108908159-108908181 | 3 | Absence |
| Intergenic:KIAA1407/ZDHHC23-KIAA1407 | chr3:113691489-113691511 | 4 | Absence |
| Intron:LIMCH1 | chr4:41,454,676-41,454,698 | 4 | Absence |
| Intron:STX18-AS1 | chr4:4,691,392-4,691,414 | 4 | Absence |
| Intergenic:RP11-724M22.1-TSPAN5 | chr4:99437910-99437932 | 4 | Absence |
| Intergenic:RP11-419L4.1-RP11-519M16.1 | chr4:130509284-130509306: | 4 | Absence |
| Intergenic:RP11-580J4.1-RP11-563E2.2 | chr4:163930772-163930794 | 4 | Absence |
| Intergenic:RP11-84A1.3-RPL21P47 | chr4:63035653-63035675 | 4 | Absence |
| Intron:C4orf19 | chr4:37547044-37547066 | 3 | Absence |
| Intergenic:AC006499.7-AC006499.6 | chr4:10200721-10200743 | 4 | Absence |
| Intron:SLC1A3 | chr5:36,613,659-36,613,681 | 4 | Absence |
| Intergenic:RP11-428C6.2-CTD-2292M14.1 | chr5:73371940-73371962 | 4 | Absence |
| Intergenic:SPINK9-RP11-373N22.3 | chr5:147741832-147741854 | 4 | Absence |
| Intergenic:C5orf63-MRPS5P3 | chr5:126471820-126471842 | 4 | Absence |
| Intergenic:snoU13-CTD-2131I18.1 | chr5:133232383-133232405 | 4 | Absence |
| Intergenic:KCNK5-KCNK17 | chr6:39,250,086-39,250,108 | 4 | Absence |
| Intergenic:SUMO2P1-MOG | chr6:29,607,587-29,607,609 | 4 | Absence |
| Intergenic:SUMO2P1-MOG | chr6_apd_hap1:911,118-911,140 | 4 | Absence |
| Intergenic:SUMO2P1-MOG | chr6_cox_hap2:1,126,393-1,126,415 | 4 | Absence |
| Intergenic:SUMO2P1-MOG | chr6_dbb_hap3:910,942-910,964 | 4 | Absence |
| Intergenic:SUMO2P1-MOG | chr6_dbb_hap3:910,942-910,964 | 4 | Absence |
| Intergenic:SUMO2P1-MOG | chr6_mcf_hap5:910,785-910,807 | 4 | Absence |
| Intergenic:SUMO2P1-MOG | chr6_qbl_hap6:910,908-910,930 | 4 | Absence |
| Intergenic:SUMO2P1-MOG | chr6_ssto_hap7:948,145-948,167 | 4 | Absence |
| Intergenic:HLA-T-DDX39BP1 | chr6_dbb_hap3:1,161,881-1,161,903 | 4 | Absence |
| Intergenic:HLA-T-DDX39BP1 | chr6_mcf_hap5:1,161,518-1,161,540 | 4 | Absence |
| Intergenic:RNU6-475P-RP11-126M14.1 | chr6:115519843-115519865 | 4 | Absence |
| Intron:TSC22D4 | chr7:100070270-100070292 | 4 | Absence |
| Intergenic:TAS2R2P-AC005281.1 | chr7:12533502-12533524 | 4 | Absence |
| Intergenic:MYL10-CUX1 | chr7:101287114-101287136 | 4 | Absence |
| Intron:PTPRN2 | chr7:158336776-158336798 | 4 | Absence |
| Intron:ZC3H3 | chr8:144609338-144609360 | 4 | Absence |
| Intron:KCNQ3 | chr8:133364200-133364222 | 4 | Absence |
| Intron:VPS13B | chr8:100865161-100865183 | 4 | Absence |
| Intergenic:CTD-2281E23.1-AF067845.1 | chr8:1267846-1267868 | 4 | Absence |
| Intergenic:RP11-383M4.6-SPATA31D1 | chr9:84592661-84592683 | 4 | Absence |
| Exon:DDX31 | chr9:135493739-135493761 | 4 | Absence |
| Intron:TLE4 | chr9:82297433-82297455 | 4 | Absence |
| Intergenic:KCNK18-RP11-501J20.5 | chr10:118,976,441-118,976,463 | 4 | Absence |
| Exon:DLG5 | chr10:79567588-79567610 | 4 | Absence |
| Intron:MUC5AC | chr11:1156835-1156857 | 4 | Absence |
| Intron:PC | chr11:66642469-66642491 | 4 | Absence |
| Intron:MUS81 | chr11:65630332-65630354 | 4 | Absence |
| Exon:RN7SL652P | chr11:47579232-47579254 | 4 | Absence |
| Exon:RP11-167N4.2 | chr11:73675522-73675544: | 4 | Absence |
| Intron:SLC38A4 | chr12:47174274-47174296 | 4 | Absence |
| Intergenic:NCOR2-SCARB1 | chr12:125239601-125239623 | 4 | Absence |
| Intergenic:UBE2D3P4-MTND4P1 | chr13:85,009,998-85,010,020 | 4 | Absence |
| Intergenic:CDKN2AIPNLP3-AZU1P1 | chr13:40399805-40399827: | 3 | Absence |
| Intergenic:LGMNP1-STARP1 | chr13:65632298-65632320: | 4 | Absence |
| Intergenic:TUBBP8-RP11-680F8.1 | chr15:29908128-29908150 | 4 | Absence |
| Intergenic:CTD-2147F2.2-CTD-2147F2.1 | chr15:97946024-97946046 | 4 | Absence |
| Intron:RP11-321G12.1 | chr15:63707118-63707140 | 4 | Absence |
| Intergenic:AGBL1-RP11-133L19.2 | chr15:87227556-87227578 | 4 | Absence |
| Exon:BANP | chr16:87,990,503-87,990,525 | 4 | Absence |
| Intergenic:CASKIN1-MLST8 | chr16:2247810-2247832: | 4 | Absence |
| Intergenic:AC092377.1-RP11-118F19.1 | chr16:85532758-85532780: | 4 | Absence |
| Exon:CES2 | chr16:66969553-66969575 | 4 | Absence |
| Intron:SDK2 | chr17:71,345,496-71,345,518 | 4 | Absence |
| Intron:STXBP4 | chr17:53235862-53235884 | 4 | Absence |
| Exon:ACACA | chr17:35608930-35608952 | 4 | Absence |
| Intron:RNF152 | chr18:59555020-59555042 | 4 | Absence |
| Intron:DEFB121 | chr20:29,998,552-29,998,574 | 4 | Absence |
| Intron:RP5-1164C1.2 | chr20:4502032-4502054 | 4 | Absence |
| Exon:DBNDD2/SYS1-DBNDD2 | chr20:44036781-44036803 | 4 | Absence |
| Intron:PROKR2 | chr20:5296251-5296273 | 4 | Absence |
| Intron:TRAPPC10 | chr21:45439747-45439769 | 4 | Absence |
| Intron:CDC45 | chr22:19,505,423-19,505,445 | 4 | Absence |
| Intergenic:CENPM-LINC00634 | chr22:42348114-42348136: | 4 | Absence |
| Intergenic:CBX7/COX5BP7-CBX7 | chr22:39524886-39524908 | 4 | Absence |
| Intergenic:Y_RNA-RP4-635G19.1 | chrX:102439911-102439933 | 4 | Absence |
| Intergenic:RP11-363G10.2-XAGE2B | chrX:52030731-52030753 | 4 | Absence |
| Intergenic:STS-VCX | chrX:7742950-7742972 | 4 | Absence |
| Intron:TMEM255A | chrX:119399285-119399307 | 4 | Absence |
| Intron:PNCK | chrX:152950907-152950929 | 4 | Absence |

! number of Nucleotide mismatched between gRNA and off-target site.
